# *In Vivo *Anti Cancer Potential of Pyrogallol in Murine Model of Colon Cancer

**DOI:** 10.31557/APJCP.2019.20.9.2645

**Published:** 2019

**Authors:** Seemaisamy Revathi, Faruck Lukmanul Hakkim, Neelamegam Ramesh Kumar, Hamid A Bakshi, Alagar Yadav Sangilimuthu, Murtaza M Tambuwala, Mohammad Changez, Mohamed M Nasef, Muthukalingan Krishnan, Nagarajan Kayalvizhi

**Affiliations:** 1 *Department of Zoology, Periyar University, Salem 636 011,*; 4 *Insect Molecular Biology Laboratory, Department of Environmental Biotechnology, Bharathidasan University, Tiruchirappalli,*; 7 *Department of Biotechnology, Karpagam Academy of Higher Education, Coimbatore, Tamil Nadu, India,*; 2 *Department of Mathematics and Sciences, College of Arts and Applied Sciences,*; 3 *Research Center, Dhofar University, Salalah, *; 8 *Chemistry Division, Department of Basic Sciences, College of Applied and Health Sciences, Sharqiyah University, Ibra, Oman, *; 5 *School of Pharmacy and Pharmaceutical Sciences, Saad Centre for Pharmacy and Diabetes, Ulster University, Cromore Road, Coleraine, Co. Londonderry, BT52 1SA, *; 6 *Department of Pharmacy, School of Applied Sciences, University of Huddersfield, Queensgate, Huddersfield, UK. *

**Keywords:** Colon cancer, Acacia nilotica, Pyrogallol, Helicobacter pylori, toxicity

## Abstract

**Background:**

Colon cancer is aggressive and it causes 0.5 million deaths per year. Practicing natural medicines for cancer treatment is safer than conventional drugs. World health organization emphasizes on the importance of practicing natural medicines and developing natural product based drugs for cancer treatment. Recently we reported an anti colon cancer activity associated with pyrogallol isolated from medicinal plant *Acacia nilotica *in HT-29 cells in vitro. To extend our observation in this study we evaluated *in vivo* colon tumor remission property of acetone extract of *A. nilotica *(ACE) and pyrogallol.

**Materials and Methods:**

*In vivo* toxicity of ACE and pyrogallol was assessed and *In vivo* tumor remission activity of ACE and pyrogallol was determined in murine model.

**Results:**

Mice were tolerated different doses of ACE and pyrogallol. Tumor size was considerably reduced in pyrogallol treated mice similar to doxorubicin. Tumor bearing mice treated with ACE and pyrogallol showed mild decline in body weight.

**Conclusion:**

Pyrogallol was found to be an effective anti colon cancer agent with less toxicity.

## Introduction

Colon cancer is the third most common type of cancer worldwide and a leading cause of cancer death. As per the American Cancer Society, there will be an estimated 145,600 new cases of colon cancer and 51,020 will die from colon cancer in 2019 (American Cancer Society, 2019). The evolution of colon cancer seems to follow a predictable pattern of histologic changes and concurrent genetic and epigenetic changes, which ultimately provide a growth advantage resulting in the clonal expansion of transformed cells (Schlussel et al., 2014). At least three forms of genomic instability contribute to colon cancer, including microsatellite instability, chromosome instability, and chromosomal translocations (Grady, 2004). A complex cellular community exists within a malignant tumor. This community was constituted by oncogenically transformed cells, non-neoplastic cells such as stromal and immune cells, and microbes such as bacteria and viruses in some cases (Kostic et al., 2012). This cellular heterogeneity is highly complex to understand and treat accordingly. Surgical resection and chemotherapy elicit significant side effects and toxicities which affects patient’s quality of life and survival. The toxicities such as hair loss, nausea, vomiting etc., drug resistance and inter-individual differences in response to any treatment are major limitations associated with current chemotherapeutic drugs (Pullarkat et al., 2001).

**Table 1:** Toxicity Associated Biochemical Parameters of Mice Treated with ACE and Pyrogallol Values are Expressed as mean ± SD (*n*=4 for each group). * indicates significant difference at p<0.05 compare to control

Parameter	Control	ACE (mg/kg body weight)			Pyrogallol (mg/kg body weight)		
		10	20	40	10	20	40
Hemoglobin (g/dL)	13.22±0.5	13.6±0.1	13.43±0.1	13.3±0.1	13.0±0.05	12.8±0.2*	12.4±0.0*
RBC (million/mm^3^)	5.1±0.1	5.05±0.1	5.01±0.3	4.96±0.5	4.9±0.5	4.86±0.6*	4.61±0.6*
WBC (T/mm3)	4.03±0.6	4.0±0.1	3.9±0.2	3.8±0.2	4.1±0.3	3.98±0.2	3.97±0.2
Neutrophils	35±0.6	35±0.2	35±0.2	35±0.1	35±0.1	35±0.1	35±0.2
Monocytes	3.5±0.1	3.5±0.1	3.5±0.1	3.5±0.5	3.5±0.5	3.5±0.2	3.5±0.1
Lymphocytes	55±0.3	55±0.1	55±0.4	55±0.1	55±0.8	55±0.7	55±0.8
Eosinophils	0.2±0.3	0.2±0.1	0.2±0.4	0.2±0.7	0.2±0.1	0.2±0.6	0.2±0.6
Basophils	0±0.05	0±0.05	0±0.05	0±0.05	0±0.05	0±0.05	0±0.05
Platelets (lakh/mm^3^)	1.63±0.3	1.63±0.6	1.69±0.2	1.71±0.1	1.74±0.5	1.75±0.5	1.77±0.5*
Cholesterol (mg/dL)	110 ±0.1	111 ±0.5	111 ±0.3	112 ±0.4	108 ±0.5	105 ±0.1	102 ±0.5
Uric Acid (mg/dL)	6.3±0.3	6.5±0.1	6.4±0.8	6.6±0.7	6.0±0.1	6.2±0.3	6.5±0.3
Alkaline Phosphatase (IU/L)	63±0.0	63±0.5	62.8±0.0	61.8±0.6	63.8±0.5	63.5±0.1	62.1±0.5
Total Protein (gm/dL)	6.3±0. 3	6.28±0. 1	6.23±0. 3	6.19±0. 5	6.3±0. 0	6.22±0. 3	6.27±0. 3
Triglyceride (mg/dL)	55±0.7	59±0.5	59±0.8	60±0.1	62±0.5	63±0.7*	65±0.1*
BUN (mg/dL)	12.6±0.0	12.4±0.6	12.3±0.1	12.2±0.5	12.9±0.1	13.2±0.5	13.7±0.8*
SGOT (IU/L)	16.3±0.5	16.1±0.4	16.2±0.2	16.0±0.5	16.9±0.5	17.3±0.2	19.3±0.0*
SGPT (IU/L)	10.6± 0.6	10.9± 0.1	11.2± 0.5	11.6± 0.5	10.6± 0.6	10.1± 0.0	10.0± 0.5

**Figure 1:**
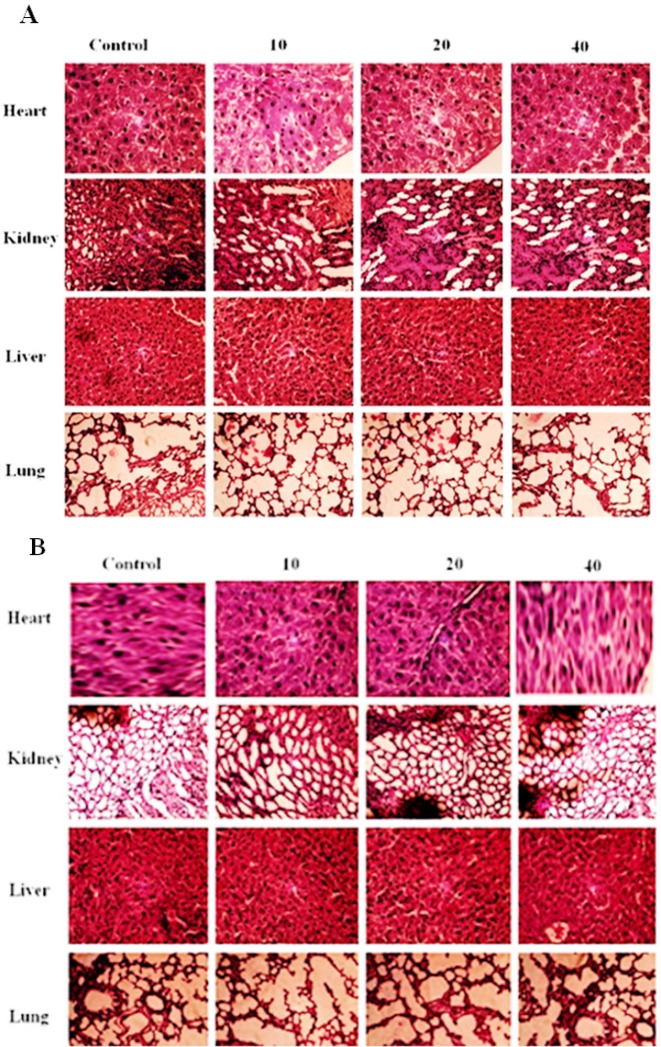
Histopathological Analysis of Heart, Kidney, Liver and Lung of the Mice Mice were Treated with Different Concentration of ACE (A) and Pyrogallol (B). 200 μm sections of heart, kidney, liver and lung were stained with H&E after treatment period. The images were photographed at 20X. No adverse side effects/changes were observed

**Figure 2:**
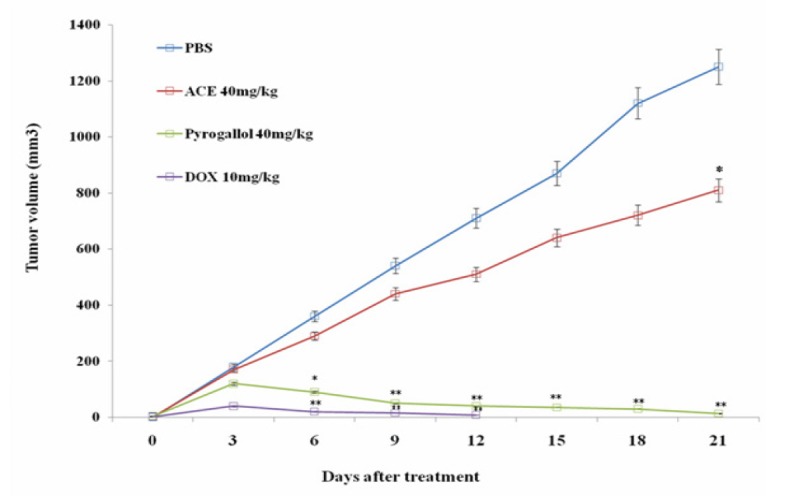
Graphical Representation of Tumor Inhibition in Mice in a Time Dependent Manner. Tumor growth curves of four diﬀerent groups of mice (4 mice per group) treated with pyrogallol and ACE The tumor volumes were measured for each group and the data are presented as mean ± SD. *, ** indicates significant difference at p<0.05 and p<0.01 compare to phosphate buffer saline (PBS)

**Figure 3:**
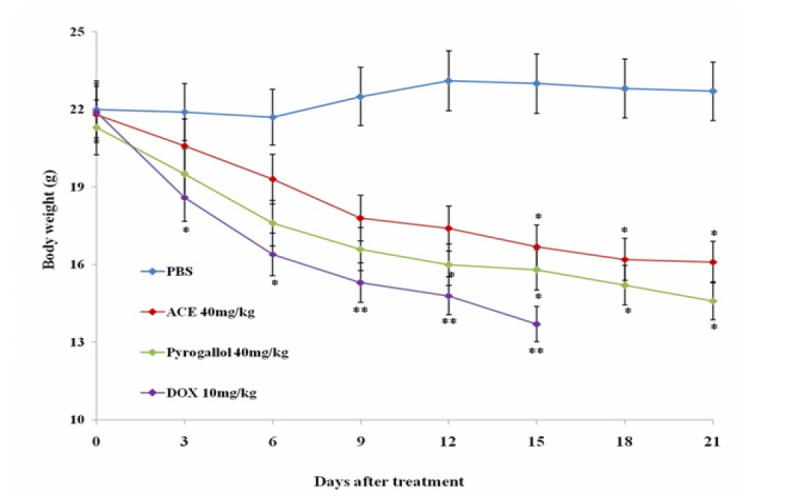
Effect of Treatment with Pyrogallol and ACE on Body Weight of Tumor bearing Mice Tumor bearing Mice were Treated with Different Concentrations of ACE and Pyrogallol. Body weight was measured for 21 days with intervals of 3 days. Data are presented as Mean ± SD. *, ** indicates significant difference at p<0.05 and p<0.01 compare to phosphate buffer saline (PBS).

In a healthy gut, the normal bacterial flora maintains homeostasis with the host (Ley et al., 2006). However, changes in bacterial populations and their metabolic products have been linked to several diseases including ulcerative colitis, crohn’s disease and colon cancer (Kaur et al., 2011; Marchesi et al., 2011; Sasaki and Klapproth, 2012). Studies suggest that the presence of certain bacteria may contribute to both the induction of inflammation in inflammatory bowel disease (IBD) and the progression of inflammation to neoplasia (Erdman et al., 2003a; Chu et al., 2004; Maggio-Price et al., 2005). Helicobacter organisms have been linked to IBD (Burich et al., 2001; Jiang et al., 2002), as well as liver cancer (Sipowicz et al., 1997), colon cancer in mice (Engle et al., 2002; Erdman et al., 2003a; Erdman et al., 2003b), and gastric cancers in humans (Uemura et al., 2002). It is evaluated that there are 1014 cell number of bacterial species present in human gastrointestinal tract, which is more than 10-fold the number of eukaryotic human cells (Ley et al. 2006) and perhaps as many as 10-fold more viruses. Natural products have been used worldwide as traditional medicines for thousands of years to treat various forms of diseases including cancer. Several studies have revealed that natural products exhibit an extensive spectrum of biological activities such as, stimulation of the immune system, antibacterial, antiviral, anti-hepatotoxic, anti-ulcer, anti-inflammatory, antioxidant, anti-mutagenic, and anti-cancer effects (Miyata, 2007; Espin et al., 2007). Pyrogallol is a natural product, a catechin type compound approved by Food and Drug Administration (FDA) as colour additive. Pyrogallol is used in the manufacturing of pharmaceuticals and pesticides and has been used for medicinal purposes as a topical antipsoriatic medicine (Willsteed and Regan, 1985). Pyrogallol reported as an anti-lung cancer agent by arresting cell cycle at G2-M phase in lung cancer cells and reduces lung tumor in animal model (Han et al., 2008; Yang et al., 2009). Furthermore, pyrogallol was shown to be an effective compound against gastric cancer in vitro (Park et al., 2008). Our previous study showed that pyrogallol isolated from *Acacia nilotica *can restrict colon cancer cell proliferation (in vitro) by modulating the cell cycle, inducing apoptosis and genotoxicity (Revathi et al., 2018). However in vitro model has numerous limitations such as the lack of tumour cellular network and their associated signaling. To check the efficacy of anti-colon cancer effect of pyrogallol *in vivo* studies are warranted. *In vivo* studies in animal models have been viewed as critical tools necessary to study the molecular mechanisms of colorectal carcinogenesis, to test potential preventative and therapeutic strategies, and to translate hypotheses derived from cell models into the complex physiology of the colon (Johnson and Fleet, 2013). As per the above statement to delineate the role of pyrogallol on colon cancer in this study we induced colon cancer by inoculating H. pylori in mice and evaluated the anti-tumour efficacy of pyrogallol. Furthermore, *in vivo* pre clinical toxicity of pyrogallol was assessed including checking the histology of major tissues, body weight and complete blood count and blood biochemical parameters.

## Materials and Methods

*Plant material*

The fresh leaves of *A. nilotica *were collected from the Foundation for Revitalization of Local Health Traditions, Bangalore, India (Latitude 12.9715987; Longitude 77.5945627). The plant was taxonomically identified by botanist and deposited in herbarium of Periyar University. 

*Preparation of A. nilotica extract (ACE)*

Extraction was carried out as described earlier (Revathi et al., 2017). Briefly, fresh leaves of *A. nilotica *was collected and washed in tap water. It was shade dried for 10 days and made into a fine powder of 40 mesh in size using the laboratory mill. 100 gm of powder was filled in a clean flat-bottomed glass container and soaked in 70% acetone for 72 h. The container with its content was sealed and kept for a period of three days accompanying occasional shaking and stirring. Extract was filtered using whatman no. 1 filter paper and it was dried at room temperature.

*Isolation and characterization of pyrogallol*

ACE was fractionated by column chromatography using different proportion of ethyl acetate and hexane and fractions were subjected to HPLC and pyrogallol was isolated based on retention time and absorbance of reference standard pyrogallol (Sigma Aldrich, Louis MO, USA). Further structure of pyrogallol was elucidated by spectral analysis such as FTIR, 1H NMR, 13CNMR, GCMS, LCMS and XRD.

*Evaluation of in vivo toxicity*

*In vivo* acute toxicity was evaluated according to the OECD guidelines (OECD, 2001). Healthy young, non-pregnant, nulliparous Swiss albino mice, weighting about 25-28 gm (8 weeks old) were placed in clean polypropylene cages with access to food and water. These cages were maintained in an air-conditioned animal house at 26 ± 2°C, 65–80% relative humidity and 12 h light-dark cycle. The animals were provided with commercial mice pellet diet and deionized water. After one week acclimation, the mice were randomly divided into 7 groups, each group consisted of five female and five male mice. They were kept separately in polypropylene cages. Group I served as control and received normal saline intraperitoneally. Group II, III, and IV treated with ACE at 10, 20, and 40 mg/kg of body weight (i.p) respectively, every 2 days for the period 21 days. Group V, VI, and VII mice were injected with pyrogallol at 10, 20, and 40 mg/kg of body weight (i.p) respectively, every 2 days for the period 21 days. At the end of the experimental period the major organs (heart, liver, kidney and lungs) were excised and fixed in 4% para formaldehyde solution and made into 200 µm sections which were stained with hematoxylin and eosin (H and E) and observed under a microscope at 20X for tissue damage. Blood sample were collected and subjected to evaluation of blood cell count and biochemical parameters. All the experiments were conducted under the guidelines of Institutional Animal Ethical Committee.

*Blood biochemical parameters*

Serum was obtained by centrifugation of whole blood at 3,000 rpm for 15 min. Serum levels of creatinine, alkaline phosphatase (ALP), total protein (TP), total cholesterol; tri-glyceride (TG), uric acid, blood urea nitrogen (BUN), glutamic oxaloacetic acid (SGOT), glutamic pyruvic transaminase (SGPT) were examined. 0.1 ml of 15 gl-1 EDTA-Na was pre-added into 1 ml of blood samples and blood samples were immediately used for testing within 2 h. The blood counts, including total count of red blood cells, white blood cell (neutrophils, lymphocytes, monocytes, eosinophils, basophils), and blood platelets were analyzed. 

*H. pylori infection of xenografts in nude mice*

C57BL/6 mice infected with *Helicobacter pylori *were generously donated by the Department of Gastroenterology, Microbiology and Pathology, Chennai Medical College Hospital and Research Centre (SRM Group), Tiruchirapalli, India (Affiliated to the Tamil Nadu Dr. M. G. R. Medical University, Chennai). Animals were in the range of 9 weeks to 10-month-old including both male and female. The anti-tumour e-ciency of pyrogallol and ACE were assessed in tumor-induced mice. When the volume of the xenograft tumour reached approximately 40-60 mm^3^ the mice were randomly divided into 4 groups. Group I: Mice treated with PBS as control, Group II: Mice treated with 40 mg/kg body weight of ACE, Group III: Mice treated with 40 mg/kg body weight of pyrogallol, Group IV: Mice treated with 10 mg/kg body weight of Doxorubicin. All compounds and vehicle were administered intravenously every 2 days, and all mice were then observed for 21 days. The tumour diameters and body weight were measured every 3 days. Doxorubicin was used as positive control. All the experiments were conducted under the guidelines of Institutional Animal Ethical Committee.

*Statistical Analysis*

The data were expressed as mean ± standard deviation (n=6). The statistical software SPSS version 17.0 was used for the analysis. Analysis of variance was done to find p values. P<0.01or 0.05 was considered significant compare to control.

## Results

*Pre clinical toxicity analysis of ACE and pyrogallol*

ACE and pyrogallol at the different dose of 10, 20 mg and 40 mg/kg body weight were administered intraperitoneally. Results revealed that no treatment related toxic symptom or mortality was observed. In addition there was no adverse effect on the behavioral responses of the tested mice at all doses examined. Physical observations indicated no signs of changes in the skin, fur, eyes mucous membrane, behavior patterns, tremors, salivation, and diarrhea of the mice. Further examination showed that there was no significant change in the hematological and biochemical parameters in the ACE and pyrogallol treated group as compared to the normal control group (Table 1). Mice treated with pyrogallol (40mg/kg of body weight) showed significant decrease in the level of hemoglobin (p<0.05), RBC count (p<0.05). The level of BUN (p<0.05) and SGOT (p<0.05) was significantly increased in mice treated with pyrogallol at 40mg/kg of body weight (Table 1). Hematoxylin and eosin staining of major organs (heart, kidney, liver, and lungs) revealed no significant differences in ACE and pyrogallol treated mice compared to control (Figure 1A and B). Liver showed normal hepatic lobular architecture, intact central vein with trapped red blood cells in a liver section from ACE and pyrogallol treated animals. The kidney revealed normal glomeruli, proximal and distal tubules, interstitium, and blood vessels. The lung tissue showed normal alveoli and heart muscle showed normal morphology compared to control (Figure 1A and B). No abnormal gross findings were observed in the necropsies of any of the mice. The food and water consumptions of the treated mice, which were measured throughout the study, were also not significantly different compared to the control mice (data not shown). 

*Anti-tumour effect of ACE and pyrogallol in H. pylori-induced colon cancer nude mice model*

Anti-tumour effect of ACE and pyrogallol was compared with doxorubicin using H. pylori induced colon cancer model. Interestingly, administration of pyrogallol at 40 mg/kg of body weight significantly reduced the tumour size about 96% in a time-dependent manner similar to that of doxorubicin (Figure 2). Whereas, in ACE treated mice tumour continued to grow similar to that in control group. There was mild change in the body weight observed in ACE and pyrogallol treated mice, however, it is better than doxorubicin treated mice (Figure 3). The results clearly suggest that ACE and pyrogallol treatment were better tolerated by *in vivo* system. Noticeably our data states that pyrogallol is an effective anti colon tumour agent with less toxicity in comparison with doxorubicin.

## Discussion

Cancer is the most deadly disease and its incidence rate enhanced every year. Unfortunately currently available chemotherapy drugs elicit intolerable toxicities which impacts on patient’s quality of life and survival (Mc Cleary et al., 2013). Surgical resection is promising treatment option for colon cancer treatment however; post surgical clinical consequences are remain unresolved. World health organization (WHO) emphasis the importance of practicing natural medicines to treat different life threatening ailments including cancer (Cheuka et al., 2017). Plant derived products such as vincristine, vinblastine, taxol etc. have contributed significantly in treating different types of tumours (Demain and Vaishnav, 2011). However, drug resistance and recurrence of cancer always are alarming factors and would necessitate discovery of new drugs which can overcome post treatment clinical complications with a better quality of life for patients.

In this study, we explored the anti-cancer activity of acetone extract of *Acacia nilotica *(*ACE*) and its active component, pyrogallol. Recently we isolated pyrogallol from ACE and studied the in vitro anti colon cancer activity. In comparison with ACE, pyrogallol showed profound in vitro anti-cancer activity against HT-29 cells by sparing normal colon cells (CRL-1831) (Revathi et al., 2018). Further we reported that pyrogallol is a genotoxic agent and induced apoptosis in colon cancer cells by modulating cell cycle progression and inhibition of migration in HT-29 cells (Revathi et al., 2018). However these in vitro evidences are not enough to reach concrete conclusions because in vitro models do not mimic the *in vivo* system. Tumour development and progression in *in vivo* is entirely different scenario since tumour cellular network is highly complex and achieving reproducibility in *in vivo* system based on in vitro data is not guaranteed. It is well known that *in vivo* studies in animal models have been viewed as critical tools necessary to study the therapeutic strategies, to test potential compounds and investigate molecular mechanisms of colon carcinogenesis. This will enable to translate hypotheses derived from cell models into the complex physiology of the colon (Johnson and Fleet, 2013). 

In this study, we developed *in vivo* colon tumour model by inoculating *Helicobacter pylori* in to mice as described earlier. To determine the effective and tolerance dose of ACE and pyrogallol for *in vivo* anti tumour experiment, we treated the mice with different concentrations of ACE and pyrogallol (10, 20 and 40 mg/kg body weight) every 2 days and observed for changes in behaviours, mortality and histology of vital organs (heart, kidney, liver, lungs) for damage (Figure 1). Further, blood cell counts and the blood biochemical parameters were analyzed in ACE and pyrogallol treated mice. Both total blood cell RBC, WBC’s (neutrophils, monocytes, lymphocytes, eosinophils, basophils) and platelets counts and levels of biochemical parameters (cholesterol, uric acid, alkaline phosphatase, total protein, triglyceride, urea nitrogen, SGOT, and SGPT) did not changed significantly following ACE and pyrogallol treatments (Table 1). The results clearly show that mice can tolerate the doses of ACE and pyrogallol, which were tested and these can be considered as safe. Similarly natural products isolated from medicinal plants reported as biocompatible agents (Peng et al., 2015; Yusuf et al., 2018).

International Agency for Research on Cancer (IARC) classified H. pylori as type 1 human carcinogen (Schistosomes, 1994). H. pylori is an infectious organism that causes colon cancer associated with infections (De Martel et al., 2012). It is well reported that increased risk of colonic neopla¬sia in individuals infected with H. pylori is associated with the rise of production of gastrin in the system triggered by this bacte¬ria (Georgopoulos et al., 2006; Sonnenberg and Genta, 2013). It is well known that oxidative stress resulting from H. pylori infection contributes to carcinogenesis (Butcher et al., 2017). Agent which can scavenge H. pylori infection mediated reactive oxygen species could halt the carcinogenesis. Previously we have shown that pyrogallol restricted the in vitro growth of H. pylori (Revathi et al., 2018). The molecular mechanism of anti H. pylori efficacy of pyrogallol is not clear however it can be suggested that pyrogallol can alleviate infection of H. pylori by interfering with their cell cycle. In this study, tumor bearing mice treated with ACE and pyrogallol and observed for reduction in tumor burden. Interestingly, pyrogallol reduces the tumour size at each time interval significantly, similar to doxorubicin (Figure 3). The molecular mechanism of H. pylori infection induced colon tumor remission property of pyrogallol is unclear. However, it has been suggested that inhibition of H. pylori induced gastric inflammation associated with oxidative stress is considered as one of the promising approaches to prevent carcinogenesis (Cao et al., 2007). This statement is in agreement with our results where pyrogallol is an antioxidant (Kang et al., 2011; Li, 2012) and reported to be an anti tumor agent in breast cancer and neuroblastoma model (Nemec et al., 2016; Sereia et al., 2019). Our current data suggests that anti colon tumor efficacy of pyrogallol could be due to scavenging of free radicals produced by H. pylori induced inflammation associated carcinogenesis in mice. Further Cao et al. reported that canolol, an antioxidant prevent the incidence of H. pylori associated gastric adenocarcinomas by scavenging oxygen radicals and reducing the levels of cyclooxygenase-2 (COX-2), inducible nitric oxide synthase (iNOS), and serum 8-hydroxy-2’-deoxyguanosine (8-OHdG) in gerbils (Cao et al., 2008). Further experiment is warranted to delineate the antioxidant role of pyrogallol in colon cancer model (*in vivo*). 

As chemotherapeutics elicit significant toxicities in patients and reflects on reduction of body weight, in our study body weight of tumor bearing mice treated with ACE and pyrogallol were measured in comparison with control group. Administration of ACE and pyrogallol did not decline the body weight of mice compared to doxorubicin treated mice. ACE and pyrogallol treatment found to be safe, however extensive preclinical toxicity studies are required to reach definite conclusion.

In conclusion, pyrogallol is an antioxidant molecule, profoundly reported for anti cancer activity. In this study, we delineate the *in vivo* anti colon cancer property of pyrogallol. Pyrogallol was found to be better tolerated by mice. H. pylori infection induced colon cancer model was developed and treated with ACE and pyrogallol. Tumor bearing mice treated with pyrogallol significantly reduced the tumor size in comparison to doxorubicin (positive control). Further tumor bearing mice received pyrogallol did not show any signs of toxicity in terms of loss of body weight, major organ damage, blood cell count and fluctuation of blood biochemical parameters. However, the molecular mechanism of *in vivo* anti colon cancer activity of pyrogallol should be studied in detail and further extensive preclinical studies is required to translate this formulation for future clinical trial.
